# Expanding the Potential Therapeutic Options for Remote Ischemic Preconditioning: Use in Multiple Sclerosis

**DOI:** 10.3389/fneur.2018.00475

**Published:** 2018-06-19

**Authors:** Carlos R. Camara-Lemarroy, Luanne Metz, Eric E. Smith, Jeff F. Dunn, V. Wee Yong

**Affiliations:** ^1^Department of Clinical Neurosciences, Cumming School of Medicine, University of Calgary, Calgary, AB, Canada; ^2^Hotchkiss Brain Institute, Cumming School of Medicine, University of Calgary, Calgary, AB, Canada; ^3^UANL School of Medicine and University Hospital, Monterrey, Mexico

**Keywords:** multiple sclerosis, ischemic preconditioning, remote ischemic preconditioning, neuroprotection, neuroimmunology

## Ischemia-reperfusion and ischemic preconditioning

We found the recent review paper by Chen et al. (1) on remote ischemic-preconditioning (ReIP) fascinating. They describe ReIP as an attractive low-cost, low-risk therapy for ischemia. But why stop there?

Ischemia-reperfusion, characterized by cascade of deleterious biochemical processes that increase target organ/systemic injury, is involved in the pathophysiology of stroke, myocardial infarction, and solid organ transplantation. Over 3 decades ago, investigators found that experimentally inducing short periods of cyclical tissue ischemia confer subsequent protection against ischemia-reperfusion-injury. This became known as Ischemic-Preconditioning (2–4). The rationale behind ischemic-preconditioning is that these short periods of ischemia do not cause irreversible injury, but instead induce an endogenous protective environment. Although playing out at different time periods, the concept is reminiscent of acclimatization to high-altitude conditions by alpinists, a process involving short term exposure to gradually increasing altitudes that induce endogenous mechanisms optimizing oxygen metabolism and conditioning for an eventual full-scale ascent (5).

The mechanisms involved in ischemic-preconditioning are complex. Beneficial changes in intracellular, anti-inflammatory, antioxidant pathways, and in gene expression have all been described (1, 3, 6). It has been shown to be protective in almost every solid organ ischemia-reperfusion-injury model. When investigators discovered that ischemic-preconditioning in one organ or limb could confer protection to a remote organ, this started a new era in ischemia-reperfusion research. This phenomenon was called ReIP (1).

The mechanisms involved in ReIP are also quite complex, although many are shared with standard ischemic-preconditioning (1). There is evidence for both humoral and neurogenic mechanisms as being the signal carriers that mediate protection, and the precise molecular pathways have yet to be elucidated (1, 3, 6, 7). Typical ReIP induced protection is known to occur in an early stage (2–3 h) and a late stage lasting up to 72 h (1, 3, 6). Long-term repeated ReIP has also been studied and shown that it can play its protective roles consistently; if applied once-daily for an extended period, the different phases of protection can be at play simultaneously or successively (8, 9). ReIP is less invasive (no need to access the target organs vasculature) and could theoretically protect any organ in the body. In the brain, ReIP before a local ischemic event is able to modulate cytokine production, maintain blood-brain barrier integrity, and promote the expression of protective intracellular molecules (1, 3, 6). In humans, ReIP of a limb (usually rendered transiently ischemic by a blood-pressure cuff) is a safe and tolerable procedure, and large trials evaluating its efficacy in acute coronary syndromes, heart surgery, organ transplantation, and stroke have been published (1, 4, 6). In cardiology, results have been mixed, with some evidence for organ-protection but inconsistent effects over clinically relevant outcomes (10–12). In neurology, there has been great interest in harnessing ReIP's protective properties in stroke research and in the elusive promise of clinically relevant neuroprotection (1). We will introduce ReIP as preconditioning to improve stroke outcome and introduce the opinion that the time is right to expand ReIP trials to other neurological disorders including MS treatment.

## ReIP in neurology: encouraging results

A promising clinical candidate for using ReIP is acute ischemic stroke. Conceivably, one would perform ReIP early after stroke onset in an attempt to protect from further ischemic injury. In an early study, investigators randomized 443 patients with acute ischemic stroke to receive pre-hospital ReIP or sham procedure before thrombolysis, and results showed a trend toward less tissue infarction although overall clinical and imaging endpoints were negative (13). In a recent, but smaller trial, involving 26 patients with acute ischemic stroke, ReIP showed a favorable trend toward improving clinical outcomes at 3 months as well as an increase in the circulating levels of a putative biomarker, heat shock protein-27 (14). Unfortunately, definitive results on hard clinical endpoints are lacking.

Investigators aiming to treat chronic conditions have evaluated the clinical feasibility of long term, repeated ReIP. In one such trial involving 68 patients, 300 days of consecutive, bilateral arm ReIP, led to reduced stroke recurrence in patients with intracranial artery stenosis (15). In another study, 17 patients with small-vessel ischemic disease underwent ReIP of their upper limbs 2 times per day for 1 full year, and investigators found improved middle cerebral artery hemodynamics and reduced white-matter lesion volumes (16). In a similar study, 30 patients with small-vessel disease and cognitive impairment underwent ReIP daily for a year, resulting in reduced white-matter lesion volume and improvements in cognitive testing (17).

In all trials, ReIP was found to be safe and tolerable, and long-term data suggests it can be used on a daily basis for years, and potentially in chronic, degenerative conditions. Furthermore, since many of the proposed endogenous protective mechanisms involved in ReIP are non-specific (antioxidant, anti-inflammatory, immune modulating) to ischemia-reperfusion-injury (1, 3, 6), it might prove efficacious in protecting against non-ischemic injury as well. In support, the wide ranging neuroprotective effects of ReIP have been demonstrated in various rodent models of non-ischemic injury, such as optic nerve transection (18), traumatic brain injury (19), and ketamine-induced neuronal apoptosis (20). Could there be a role for ReIP in the treatment of Multiple Sclerosis (MS)?

## ReIP in MS: some pathophysiological links

There are two main ways ReIP could be relevant in MS. On the one hand, MS pathophysiology might involve ischemic/hypoxic mechanisms, and on the other, some of the pathways beneficially modulated by ReIP could be protective against inflammatory demyelination/neurodegeneration. A recent review summarizes multiple lines of evidence suggesting that there is generalized cerebral hypo-perfusion and chronic hypoxia in patients with MS, and that this could contribute to neurodegeneration (21). Furthermore, the white-matter regions more commonly affected in demyelinating disease are similar to those affected by small-vessel disease. Oligodendrocytes are very susceptible to hypoxia and in animal models of demyelination, demyelination is prevented if adequate oxygenation is maintained (22). A recent study was able to show *in vivo* that there is reduced cortical microvascular oxygenation in patients with MS (23). Nevertheless, the precise role of hypoxia/ischemia in MS remains unknown.

There is also evidence suggesting that ReIP can modulate some of the mechanisms involved in the pathogenesis of Experimental Autoimmune Encephalomyelitis (EAE), the prototypical animal model of MS. In mice studies, hind-limb ReIP increased serum and mRNA expression of erythropoietin (EPO) and hypoxia-inducible factor-1alpha (HIF1-alpha) in the brain (24, 25). EPO is known to be neuroprotective in the mouse EAE model (26), and HIF1-alpha is increased in EAE, acting as a transcription factor for IL-17-triggered cytokine production (27). Heat shock protein (Hsp)70 is also one of the principal mediators of ReIP-induced neuroprotection, by mediating chaperone-cytoprotective effects, blocking multiple steps in the apoptosis pathway and immune-modulation (25, 28). In EAE, Hsp70 is upregulated, and Hsp70 knockdown alters the immune response associated with demyelination (29, 30). Other shared mechanisms in ReIP-induced protection and in EAE pathophysiology are the mTOR kinase (involved in regulation of immune cell function) and nitric oxide signaling (31–33).

Studies also point toward hypoxia being associated with EAE pathophysiology. Investigators found direct and indirect evidence of tissue hypoxia in the spinal cord of a rat model (34), and the cortex and cerebellum of a mouse model (35), as well as a protective effect of oxygen therapy (34). Mice kept in hypoxic conditions (hypoxic preconditioning) show decreased numbers of (CD)4+ T cells and a delayed Th17-specific cytokine response in the spinal cord after EAE induction, as well as increased numbers of Treg cells and Interleukin (IL)-10 (36). Another study found that hypoxic preconditioning delayed the onset of EAE, and when hypoxic preconditioning was established after the onset of clinical symptoms, spinal cord pathology, and inflammation decreased (37).

ReIP can also alter cellular and humoral immune responses classically thought to be key regulators of MS pathogenesis. Ischemic-preconditioning can induce an increase in CD4(+)CD25(+)FoxP3(+) and CD4(+)CD25(+)IL-10(+) Tregs in peripheral organs (38, 39). Tregs are essential to resolve immune responses in EAE (40). In mouse models of stroke, ReIP was able to modulate the populations of peripheral T and B cells (41) and inhibit the galectin-9/Tim-3 inflammatory cell signaling pathway, which induces cell death in lymphocytes (42). Resident CNS cells such as microglia and astrocytes also respond to ischemic-preconditioning by a shift in function toward provision of trophic support and neuroprotection (31). In fact, activation of inflammatory pathways may be necessary for the induction of ischemic tolerance by ReIP, eventually leading to subdued immune activation (43).

We have summarized molecular/inflammatory pathways involved in MS pathophysiology that appear to be modulated by ReIP. However, other pathways involved in ReIP-induced protection, such as autophagy, potassium channels, adenosine, and neurogenic signals are neuroprotective (1, 3, 6), and might also be of benefit in the context of inflammatory demyelination/neurodegeneration.

**Figure 1:**
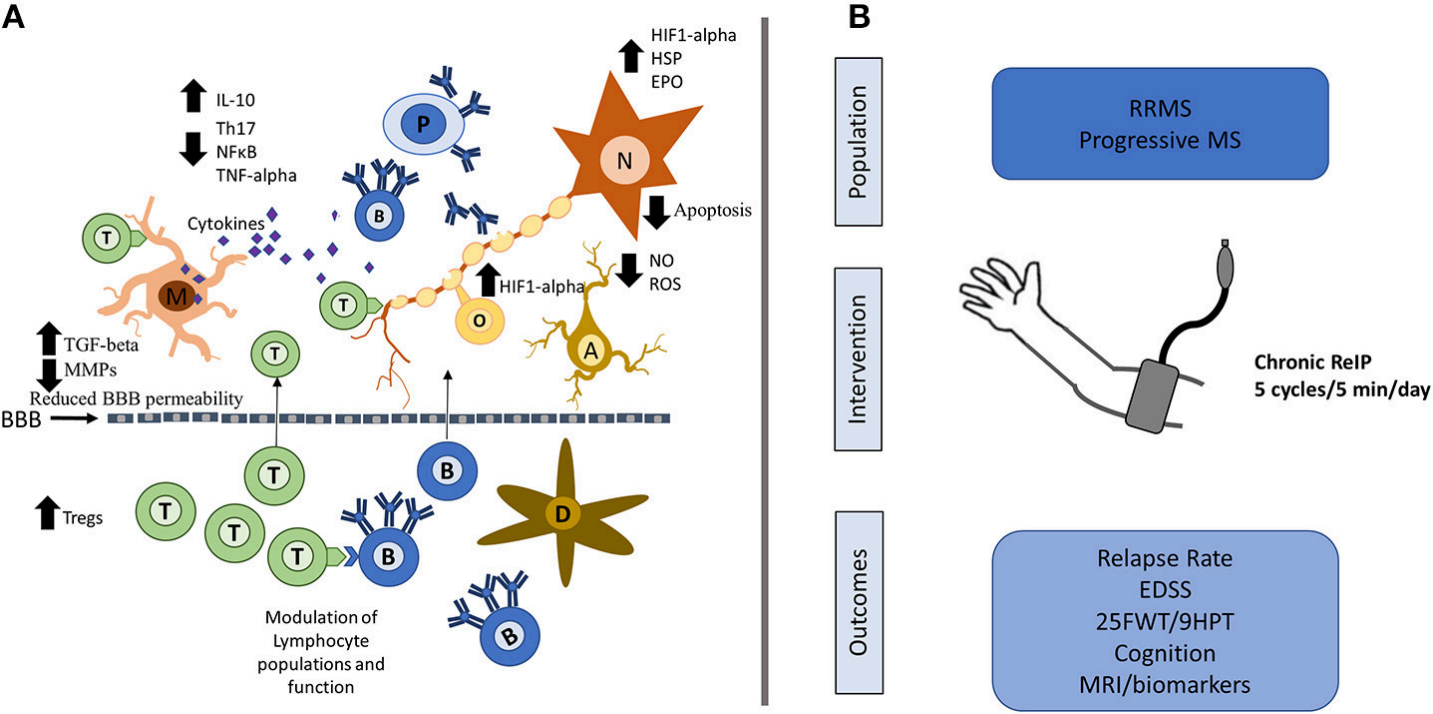
Possible neuroprotective mechanisms of ReIP in MS and proposed intervention. **(A)** ReIP can modulate several of the typical pathogenic mechanisms involved in MS. In the periphery, ReIP can alter lymphocyte populations and may also maintain blood brain barrier (BBB) permeability through reduction in matrix metalloproteinases (MMPs) and increases in transforming growth factor beta (TGF-beta). In the central nervous system, ReIP increases the expression of hypoxia inducible factor-1 alpha (HIF1-alpha), heat shock proteins (HSP) and erythropoietin (EPO), conferring neurons and glia protection against inflammatory insults and apoptosis. ReIP also reduces the production of pro-inflammatory cytokines such as tumor necrosis factor alpha (TNF-alpha) and increases levels of anti-inflammatory cytokines such as interleukin (IL)-10. ReIP ameliorates oxidative stress by reducing the production of reactive oxygen species (ROS) and nitric oxide (NO). **(B)** Patients with relapsing remitting MS (RRMS) or progressive forms of MS could be randomized to receive daily, chronic arm ReIP for 1–2 years or a sham procedure. Relevant outcomes would include relapse rate for RRMS, and for all patients, measures of disability such as the expanded disability status score (EDSS), 25 foot-walking test (25FWT), the 9-hole peg test (9HPT) and measures of cognition. MRI biomarkers looking at lesion load and atrophy as well as plausible biomarkers could be of use as well. T, T-lymphocytes; B, B-lymphocytes; D, dendritic cell; M, microglia; N, neuron; A, astrocyte; O, oligodendrocyte; P, plasma cell; NF-κB, nuclear factor kappa-B.

ReIP might then stimulate an endogenously protective milieu in the brain that could theoretically reduce inflammatory demyelination/neurodegeneration through its non-specificity (Figure 1A). Interestingly, there is evidence suggesting that the brain is already attempting to use this strategy in MS. In a pathology study on normally appearing white-matter from patients with MS, investigators found an upregulation of genes associated to ischemic-preconditioning mechanisms such as HIF-1alpha, PI3K/Akt signaling among others (44). The authors of this study suggested that these molecular changes might reflect an adaptation of cells to the chronic progressive pathophysiology of MS. In a model of cultured glia treated with Th1 and Th2 cytokines, investigators found that many of the changes in gene expression were similar to those seen in ischemic-preconditioning and EAE (genes related to mitochondrial function, neurotransmission, vitamin D metabolism, and a variety of transcription factors) (45).

Although the mechanisms involved might differ from ReIP, an exciting area of research from where further encouragement might be derived is the neuroprotective potential of hypoxic preconditioning (46, 47). In a study involving patients with chronic incomplete spinal cord injuries, a regimen of intermittent hypoxia (short term inhalation of a low-oxygen mixture through a breathing mask) was shown to increase volitional strength in specific muscle groups, albeit transiently and immediately after hypoxia (48). In a randomized, double-blind, placebo-controlled trial, 15, 90-s exposures to hypoxia for 5 days led to increased walking speed distances in patients with incomplete spinal cord injury, with a sustained effect over 2 weeks (49). A recent trial using a similar design confirmed the benefits of repetitive intermittent hypoxia in patients with incomplete spinal cord injury, and showed a sustained effect over 5 weeks in walking speed and endurance (50).

## ReIP: a proposal in MS

ReIP induces protective changes in the central nervous system; it is safe, tolerable, and can be induced for virtually indefinite periods of time; it may also ameliorate both acute inflammatory insults and chronic neurodegenerative processes. We suggest that ReIP could potentially be of therapeutic value in MS (Figure 1B). However, several issues would have to be addressed before a trial is designed. MS is a heterogeneous disease, and it is unclear what sub-population would be ideally suited for a clinical trial. Long-term functional endpoints, and cognitive measures in particular, may be realistic goals. Chronic ReIP induced in the upper extremities, such as that described in trials of cerebral small vessel disease for progressive-forms of MS may be favored. ReIP could also be conceived as an add-on therapy, with the benefit that it has no known or theorized pharmacological interactions. Imaging biomarkers, including white-matter lesion and brain atrophy measures would be ideal secondary endpoints, and putative mechanistic biomarkers such as HIF1-alpha and HSPs could be readily analyzed.

## Conclusion

Recent research on the underlying mechanisms of white-matter pathology have found curious similarities between ischemic and non-ischemic disorders (51). Furthermore, the CNS appears to use redundant, endogenous protective mechanisms against different types of insults. Even if the precise mechanism involved in ReIP-induced neuroprotection remain elusive, we are optimistic in the prospects of expanding its indications in neurology beyond ischemic conditions. There is currently one trial aiming to evaluate the safety and tolerability of ReIP in patients with MS, with the aim of showing short-term benefits on exercise tolerance (52). While we await the results of this trial with great interest, we believe that ReIP might be a way to harness these mechanisms to protect against inflammatory demyelination and associated neurodegeneration. A clinical trial will be the only way to evaluate the merits of this proposal.

## Author contribution

CC-L: conceptualization and writing of original draft; LM, JD, and ES: writing, review, and editing; VY: supervision, writing, review, and editing.

### Conflict of interest statement

The authors declare that the research was conducted in the absence of any commercial or financial relationships that could be construed as a potential conflict of interest.
